# An Investigation of the Secretome Composition of *Coriolopsis trogii* Mafic-2001 and the Optimization of the Mafic-2001 Enzyme Cocktail to Enhance the Saccharification Efficacy of Chinese Distillers’ Grains

**DOI:** 10.3390/ijms26104702

**Published:** 2025-05-14

**Authors:** Chengling Bao, Zhiyun Liu, Xiaoxia Zhong, Xiaofeng Guan, Yunhe Cao, Jinxiu Huang

**Affiliations:** 1 National Center of Technology Innovation for Pigs, Chongqing 402460, China; baochengling@163.com (C.B.); liuzhiyun2009.6@163.com (Z.L.); zhongchuck@163.com (X.Z.); ggguanxf@163.com (X.G.); 2 Chongqing Academy of Animal Science, Chongqing 402460, China; 3 State Key Laboratory of Animal Nutrition and Feeding, College of Animal Science and Technology, China Agricultural University, Beijing 100193, China; caoyh@cau.edu.cn

**Keywords:** lignocellulose, enzyme cocktail, *Coriolopsis trogii*, β-glucosidase, endoglucanase, Chinese distillers’ grains

## Abstract

The efficient degradation of lignocellulose is essential for valorizing agricultural waste and reducing environmental pollution. An efficient degradation process requires an enzyme cocktail capable of comprehensively deconstructing lignocellulosic components. In this study, the secretome of *Coriolopsis trogii* Mafic-2001 induced by rice straw was examined, and the enzymatic composition and reaction conditions of *Coriolopsis trogii* were optimized. Mafic-2001 secreted an enzyme cocktail that included ligninolytic enzymes, cellulases, and hemicellulases. However, the relative abundances of endoglucanase (EG) and β-glucosidase (βG) were only 64.37% and 10.69%, respectively, compared with the relative abundance of cellobiohydrolase, which indicated a critical bottleneck in degradation efficiency. To overcome this limitation, the recombinant enzymes rEG1 and rβG1 were expressed in *Pichia pastoris* X-33. A functionally enhanced enzyme cocktail (rEG1–rβG1–Mafic-2001 = 0.05:0.09:0.86) was developed via a mixture design to achieve a reducing sugar yield of 2.77 mg/mL from Chinese distillers’ grains (CDGs). Structural analyses revealed that the optimized enzyme cocktail disrupted the reticulated fiber architecture of CDGs and attenuated the characteristic Fourier-transform infrared spectroscopy peaks of lignin, cellulose, and hemicellulose. This study elucidates the synergistic lignocellulose deconstruction mechanism of Mafic-2001 and establishes a precision enzyme-supplementation strategy for efficient CDG bioconversion, providing a scalable platform for the valorization of lignocellulosic biomass.

## 1. Introduction

Lignocellulose is an abundant renewable resource, and its conversion into biofuels and high-value biological products is crucial for promoting resource recycling and reducing carbon emissions [1,2]. Chinese distillers’ grains (CDGs), byproducts of the Baijiu industry, possess distinctive flavor profiles and valuable nutritional components, including trace elements and proteins, which makes them a potential feed ingredient for livestock. However, strong-flavor CDGs contain a significant proportion of rice husks (one-third on a dry basis), where the high lignocellulose content in rice husks severely limits the efficient utilization of CDGs [3]. The structural complexity of lignocellulose requires the synergistic action of cellulase, hemicellulase, lignin-degrading enzymes, and various accessory proteins for complete hydrolysis. However, low enzymatic hydrolysis efficiency and the high cost of enzymes remain major bottlenecks that hinder the effective saccharification of lignocellulose [4].

White-rot fungi (WRFs) are a group of filamentous fungi capable of completely decomposing plant-derived lignocellulose, leaving behind a characteristic white residue [5]. Their high degradation efficiency and environmental compatibility have made them a key focus in lignocellulose degradation research [6]. The lignocellulose degradation mechanism of WRFs mainly relies on their abundant extracellular ligninolytic and cellulolytic enzymes, which break down lignin and cellulose biopolymers, respectively. The primary lignin-degrading enzymes include laccase (Lac), lignin peroxidase (LiP), and manganese peroxidase (MnP) [7]. WRFs are major producers of hemicellulases, and they secrete a variety of enzymes, including main-chain and side-chain enzymes such as xylanase, mannanase, galactosidase, and arabinofuranosidase [8]. While WRFs also produce cellulolytic enzymes, including cellobiohydrolase (CBH), endoglucanase (EG), and β-glucosidase (βG) [7], their cellulose hydrolysis mainly generates low-molecular-weight carbohydrates rather than fully converting cellulose into monosaccharides. This limitation arises from the absence of critical enzymatic components in their cellulase system that are necessary for complete cellulose depolymerization into monosaccharides [9].

Insufficient βG activity leads to the accumulation of cellobiose, which in turn causes the severe product feedback inhibition of EG and CBH [10]. Moreover, the limited presence of hemicellulolytic enzymes in the *Trichoderma reesei* secretome significantly hinders the efficient breakdown of lignocellulosic biomass [11]. To enhance the conversion efficiency of lignocellulosic biomass, enzyme cocktails capable of effectively degrading all components are developed [12]. An effective strategy for optimizing enzyme cocktails and improving hydrolysis efficiency involves supplementing specific enzymes based on the composition of the native enzyme cocktail, thereby enhancing synergistic interactions among different enzymes. For example, supplementing βG and EG in *Trichoderma reesei* enzyme cocktails has been shown to enhance cellulose conversion rates in sugarcane bagasse [13]. A comprehensive understanding of the secretomes of relevant microorganisms will aid in the development of highly efficient and customized in vitro lignocellulose-hydrolyzing enzyme cocktails [14].

To elucidate the protein composition of extracellular lignocellulose-degrading enzyme cocktails in WRFs, optimize the enzyme cocktail composition, and enhance the lignocellulose hydrolysis efficiency, this study focuses on *Coriolopsis trogii* (*C. trogii*) Mafic-2001, a WRF strain isolated in our laboratory. Mafic-2001 simultaneously secretes high levels of laccase, cellulases, and hemicellulases. However, the composition of its secretome and its degradation efficiency for agricultural waste remain uncharacterized. This study investigates the secretome of *C. trogii* Mafic-2001 under lignocellulosic biomass induction. Additionally, EG and βG were heterologously expressed and supplemented into the native enzyme cocktails of Mafic-2001 for the hydrolysis of CDGs. This study lays the foundation for elucidating the mechanistic roles of lignocellulose-degrading secretomes in WRFs and for developing high-efficiency enzyme cocktails. Furthermore, this study provides strategic insights for improving the utilization efficiency of agricultural waste.

## 2. Results

### 2.1. Effects of Different Lignocellulosic Biomass and Fermentation Time on the Production of Lignocellulose-Degrading Enzymes by C. trogii Mafic-2001

**Figure 1:**
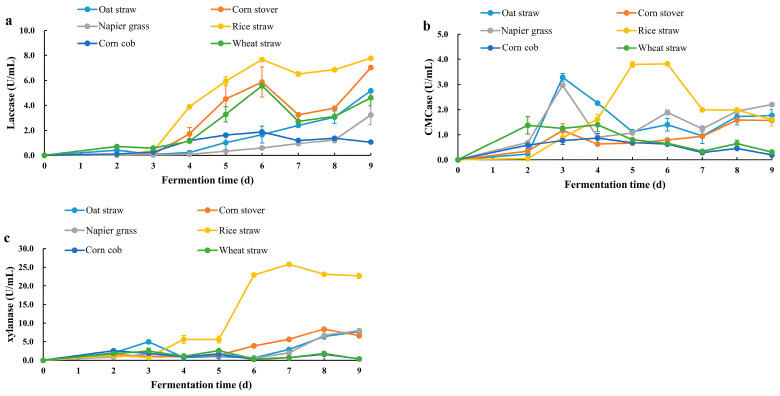
Activities of laccase, cellulase, and xylanase secreted by *C. trogii* Mafic-2001 induced by different lignocellulosic biomasses. (**a**) Laccase activity in cultures; (**b**) carboxymethyl cellulase (CMCase) activity in cultures; (**c**) xylanase activity in cultures. The results represent the mean ± SD (*N* = 3).

To prepare a lignocellulose-hydrolyzing enzyme cocktail, various lignocellulosic biomasses—including oat straw, corn stover, corn cob, Napier grass, wheat bran, and rice straw—were used as carbon sources to induce lignocellulose-degrading enzyme production in *C. trogii* Mafic-2001 (Figure 1). The activities of laccase, cellulase, and xylanase were quantified, and it was revealed that rice straw induction resulted in the highest laccase and cellulase activities after six days of cultivation (7.67 U/mL and 3.82 U/mL, respectively), while xylanase activity peaked at 25.83 U/mL on the seventh day. Notably, oat straw cultivation for three days achieved 86% of the maximum cellulase activity observed with rice straw (3.29 U/mL vs. 3.82 U/mL). Consequently, rice straw was selected as the optimal substrate for inducing lignocellulose-degrading enzyme secretion in Mafic-2001. And the use of rice straw as the substrate enabled the preparation of the lignocellulolytic enzyme cocktail (Mafic-2001).

### 2.2. Extracellular Enzymes in the Proteome of C. trogii Mafic-2001

**Figure 2:**
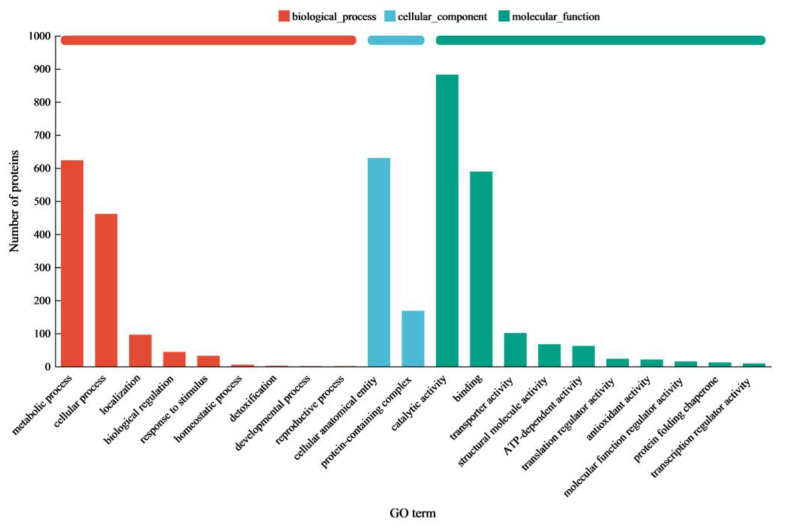
Annotation of GO functions in the proteome of *C. trogii* Mafic-2001.

**Figure 3:**
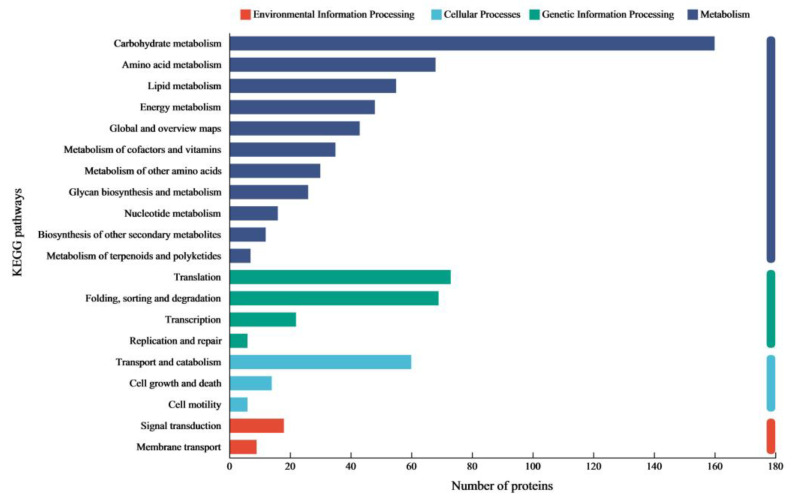
KEGG functional annotation of the proteome of *C. trogii* Mafic-2001.

To elucidate the lignocellulose degradation mechanism of the enzyme cocktail from *C. trogii* Mafic-2001, the proteome was profiled via data-independent acquisition (DIA) proteomics under rice straw induction. A total of 1443 proteins were identified, and functional annotation was performed via Gene Ontology (GO) and Kyoto Encyclopedia of Genes and Genomes (KEGG) analyses. The GO enrichment results revealed that extracellular proteins were predominantly distributed across two major categories: biological processes and molecular functions (Figure 2). Within the biological processes category, proteins were significantly enriched in carbohydrate metabolic processes and cellular processes. In the molecular functions category, primary enrichment occurred in catalytic activity and binding activity. KEGG pathway analysis, a database for metabolic network characterization, enabled a systematic exploration of the biological functions in the Mafic-2001 proteome (Figure 3). Four major metabolic categories were enriched: metabolism (56% of annotated genes), genetic information processing (27%), cellular processes (12%), and environmental information processing (5%). Among the metabolic pathways, carbohydrate, amino acid, and lipid metabolism showed the highest protein enrichment. These findings demonstrated that the proteome of Mafic-2001 held substantial potential for lignocellulose deconstruction, supported by its comprehensive enzymatic repertoire and metabolic versatility.

Carbohydrate-active enzymes (CAZymes) are mainly involved in the metabolism of carbohydrates both intracellularly and extracellularly. A total of 287 CAZyme-associated proteins were identified using the CAZy database (http://www.cazy.org, accessed on 2 January 2025) for annotation. for annotation. Glycoside hydrolases (GHs, 143 proteins) were the most abundant category, followed by auxiliary activities (AAs, 53 proteins), carbohydrate-binding modules (CBMs, 37 proteins), carbohydrate esterases (CEs, 25 proteins), glycosyltransferases (GTs, 18 proteins), and polysaccharide lyases (PLs, 11 proteins). These data indicated that lignocellulose deconstruction by *C. trogii* Mafic-2001 involved the synergistic collaboration of multiple enzyme classes. Subsequently, a comprehensive analysis of the functions and relative abundance of CAZymes was conducted to characterize the functional roles and catalytic mechanisms of these enzymatic components.

Predicted proteins in the extracellular proteome of Mafic-2001 were functionally classified using the CAZy, NR, and Swiss-Prot databases. Proteins with relative abundances of ≥0.01% were then subjected to systematic quantification. As shown in Table 1, the extracellular proteome contained 24 identified cellulases and formed an integrated cellulose hydrolysis system. This enzymatic portfolio included four CBHs (three GH7 and one GH6), nine EGs (three GH61, one GH6, two GH12, and three GH5), and eleven βGs (five GH3, three GH5, two GH55, and one GH1). The cumulative relative abundances of CBH, EG, and βG enzymes accounted for 7.58%, 4.88%, and 0.81% of the total proteome, respectively.

Under rice straw induction, *C. trogii* Mafic-2001 secreted not only a complete set of cellulose-degrading enzymes but also a diverse repertoire of hemicellulose-degrading enzymes (Table 2). The hemicellulolytic enzymes included three endo-1,4-β-xylanases (three GH10), one xyloglucanase (GH74), two β-xylosidases (one GH3 and one GH5), seven mannosidases (two GH2, two GH5, one GH76, one GH47, and one GH38), three galactosidases (one GH27 and two GH35), and three carboxylesterases (CE1). The cumulative relative abundance of hemicellulose-degrading enzymes in the proteome reached 2.11%, which highlighted their collective role in biomass deconstruction.

The ligninolytic enzymes of Mafic-2001 were identified as lignin-degrading enzymes (Table 3), including six laccases (AA1) and one manganese peroxidase (AA2). The cumulative relative abundance of laccases reached 5.45%, which highlighted their substantial ligninolytic potential. Furthermore, fourteen redox-active enzymes were identified (Table 4), including one FAD-binding oxidoreductase (0.34%), two GMC oxidoreductases (0.24%), and one lytic polysaccharide monooxygenase (LPMO, 0.27%), with higher relative abundances compared to other redox enzymes.

Pectin, a structurally heterogeneous heteropolysaccharide with a complex composition, requires coordinated enzymatic action for complete degradation. As detailed in Table 5, the pectin-degrading enzymes secreted by Mafic-2001 include one rhamnogalacturonan acetylesterase (CE12), one endo-polygalacturonase (GH28), three exopolygalacturonases (GH28), one polygalacturonase (GH28), and one α-L-rhamnosidase (GH28), with a cumulative relative abundance of 1.19% in the proteome. Furthermore, Mafic-2001 exhibited starch-degrading enzymatic activity (Table 6), including one glucoamylase (GH15) and three α-amylases (GH13), with a total relative abundance of 0.70%.

### 2.3. Heterologous Expression of EG rEG1 and βG rβG1

In this study, in order to achieve efficient expression in *Pichia pastoris* (*P. pastoris*), the genes *EG1* and *βG1* were optimized based on the codon usage bias of *P. pastoris*, without altering the amino acid sequences (Figure S1). Sequence alignment revealed that the optimized *EG1* gene (*EG1*-opt) shared 75.00% of its nucleotide identity with the wild-type sequence (*EG1*-wt), and the codon adaptation index (CAI) improved from 0.56 to 0.85 (Figure S2). Similarly, the optimized *βG1* gene (*βG1*-opt) shared 75.02% of its identity with its wild-type counterpart (*βG1*-wt), with CAI values increasing from 0.57 to 0.85 (Figure S3).

**Figure 4:**
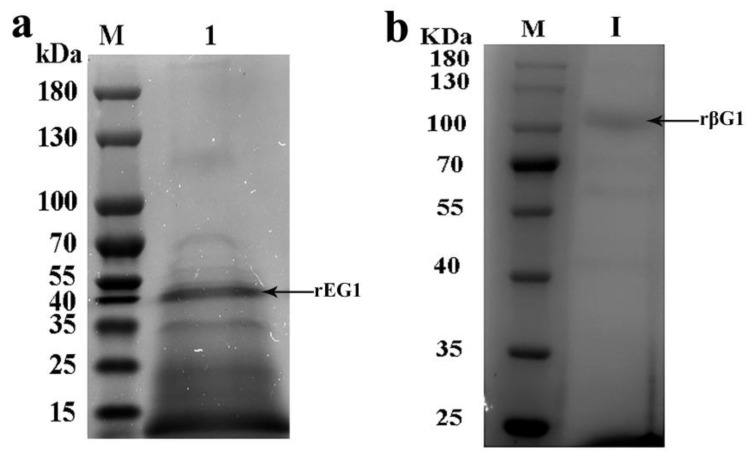
SDS-PAGE analysis of proteins expressed in recombinant strains X-33/EG1 and X-33/βG1. (**a**) Protein expressed in recombinant strain X-33/EG1. M: protein molecular marker; 1: supernatant from recombinant strain X-33/EG1 in shake flasks. (**b**) Purified protein expressed in recombinant strain X-33/βG1. M: protein molecular marker; Ⅰ: supernatant from recombinant strain X-33/βG1 in shake flasks.

Recombinant strains X-33/EG1 and X-33/βG1 were induced for methanol-driven enzyme expression in the culture medium. After 72 h of methanol induction, the extracellular cellulase and β-glucosidase activities in the supernatants of X-33/EG1 and X-33/βG1 reached 2.56 U/mL and 31.56 U/mL, respectively. The molecular weights of rEG1 and rβG1 were analyzed via sulphate–poly-acrylamide gel electrophoresis (SDS-PAGE). The analysis revealed distinct protein bands that corresponded to recombinant rEG1 and rβG1 (Figure 4), with molecular weights of approximately 45 kDa and 100 kDa, respectively (Original images are shown in Figures S4 and S5).

### 2.4. Optimization of Enzyme Cocktail Mafic-2001 and Enzymatic Hydrolysis Conditions for CDG Degradation

**Figure 5:**
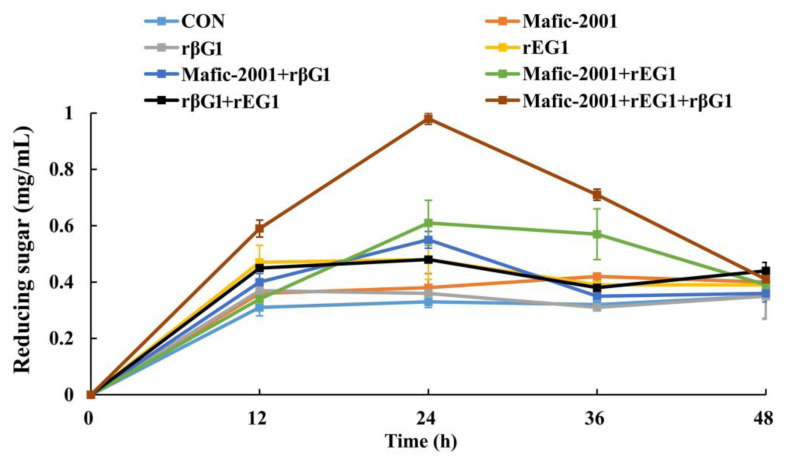
Effects of rEG1, rβG1, enzyme cocktail Mafic-2001, and their different combinations on reducing sugar yield in hydrolyzed CDGs. The results represent the mean ± SD (*N* = 3).

To enhance the degradation efficiency of lignocellulose in CDGs, recombinant cellulases (rEG1 and rβG1) derived from *C. trogii* Mafic-2001 were combined with the native enzyme cocktail Mafic-2001, which was induced by rice straw. The hydrolytic performance of various enzyme combinations on CDGs was systematically evaluated (Figure 5). The reducing sugar content produced by the hydrolysis of CDGs with different enzyme combinations (Mafic-2001 + rβG1, Mafic-2001 + rEG1, and Mafic-2001 + rEG1 + rβG1) was higher than that obtained from single enzyme treatments (Mafic-2001, rEG1, and rβG1). For instance, the Mafic-2001 + rβG1 group produced 45% more reducing sugars than Mafic-2001 alone, while the Mafic-2001 + rEG1 group yielded 61% higher reducing sugars than Mafic-2001. Notably, the ternary combination (Mafic-2001 + rEG1 + rβG1) achieved the highest reducing sugar production and demonstrated a synergistic enhancement in lignocellulose deconstruction.

**Figure 6:**
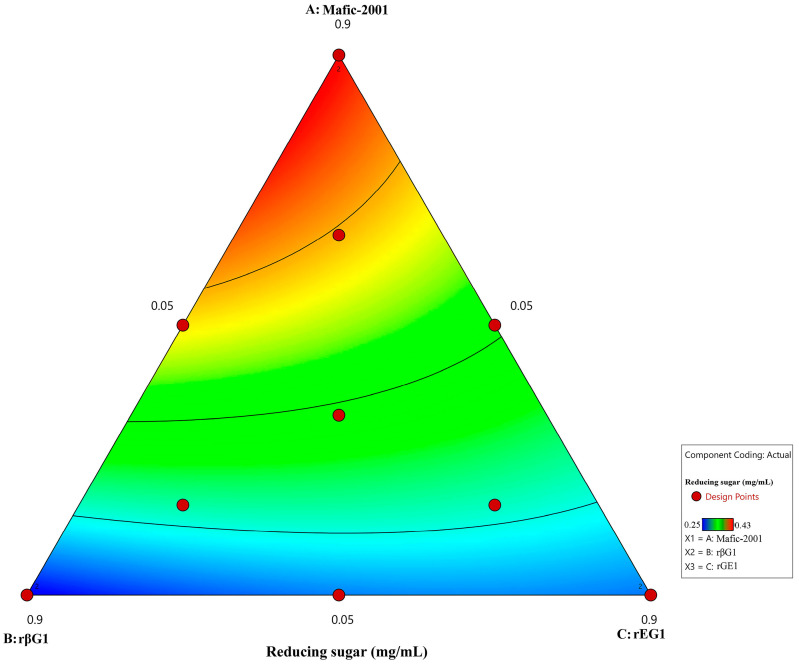
Ternary contour plot showing variations in reducing sugar yield across enzyme ratios.

The optimal ratio of recombinant enzymes rEG1 and rβG1 to the native enzyme cocktail Mafic-2001 was systematically optimized via a mixture design approach. A total protein loading of 120 mg/g was allocated to the three enzymes (recombinant cellulases rEG1 and rβG1 and the native enzyme cocktail Mafic-2001) to formulate the combinatorial enzyme cocktail (Table 7). The highest reducing sugar concentration was achieved with a ratio of rEG1–rβG1–Mafic-2001 = 0.05:0.05:0.9. Multiple regression analysis of the mixture design response values yielded the model Y = 0.43 X_1_ +0.22 X_2_ +0.26 X_1_ +0.28 X_1_X_2_ +0.02 X_1_X_3_ +0.07 X_2_X_3_, where Y represents the reducing sugar yield (mg/mL), and X_1_, X_2_, and X_3_ denote the proportions of native enzyme cocktail Mafic-2001, rβG1 and rEG1, respectively. The model identified an optimized ratio of Mafic-2001–rβG1–rEG1= 0.86:0.09:0.05. Regression coefficients revealed the relative impact of each variable. The native enzyme cocktail Mafic-2001 exhibited the highest coefficient (0.43), which indicated its dominant role in hydrolysis efficiency. Ternary contour plots derived from the regression model demonstrated that maximal hydrolysis occurred within a specific range of rEG1 and rβG1 proportions, beyond which efficiency declined. The native enzyme cocktail Mafic-2001 played a dominant role in lignocellulose deconstruction, while excessive ratios of recombinant enzymes (rEG1 and rβG1) negatively impacted synergistic activity (Figure 6). Under the optimal ratio determined by the model, validation experiments demonstrated that the optimized enzyme cocktail hydrolyzing CDGs achieved a reducing sugar yield of 0.44 mg/mL, closely aligning with the model’s predicted results.

**Figure 7:**
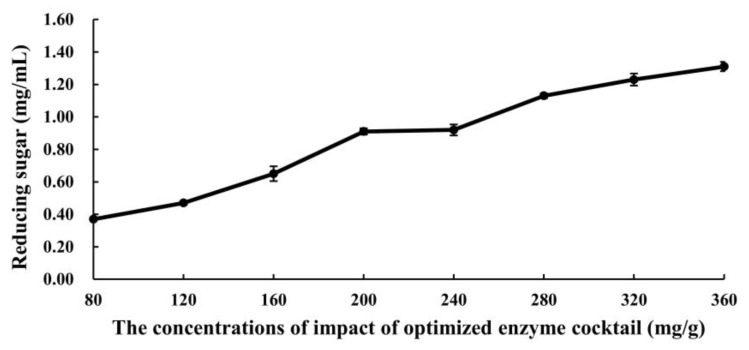
Effect of optimized enzyme cocktail concentrations on CDG hydrolysis efficiency. The results represent the mean ± SD (*N* = 3).

The hydrolysis conditions were further refined based on the optimized ratio of rEG1, rβG1, and the native enzyme cocktail Mafic-2001. The effect of optimized enzyme cocktail concentrations on CDG hydrolysis efficiency is shown in Figure 7. There was a positive correlation between the enzyme concentration and reducing sugar yield, with the rate of increase gradually diminishing at higher concentrations. When enzyme loading exceeded 280 mg/g, a notable deceleration in reducing sugar production was observed despite continued incremental gains. This kinetic pattern suggests the potential saturation of the enzyme–substrate system, where the maximal binding capacity between the catalytic sites and available substrate may have been approached. Under such saturation conditions, additional enzyme supplementation fails to proportionally enhance hydrolytic efficiency due to substrate limitation and potential mass transfer constraints while simultaneously incurring elevated operational costs through excessive enzyme utilization. According to a cost–benefit analysis, which balanced hydrolysis efficiency and economic viability, 320 mg/g was identified as the optimal enzyme loading.

**Figure 8:**
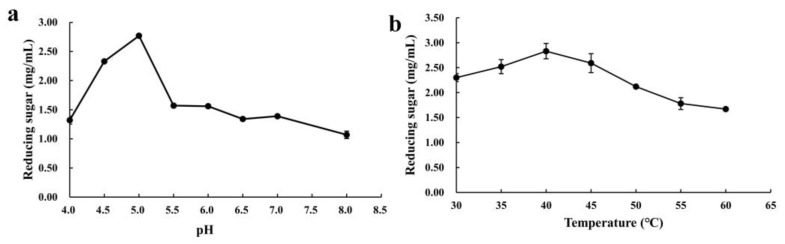
Effects of the optimized enzyme cocktail treatment on reducing sugar yield in hydrolyzed CDGs. (**a**) Effects of different pH levels on reducing sugar yield in CDGs. (**b**) Effects of different temperatures on reducing sugar yield in CDGs. The results represent the mean ± SD (*N* = 3).

Furthermore, the effects of pH and temperature on the hydrolysis efficiency of the optimized enzyme cocktail toward CDGs were investigated (Figure 8). The optimized enzyme cocktail exhibited maximal hydrolytic activity at pH 5.0, which resulted in a reducing sugar concentration of 2.77 mg/mL (Figure 8a). The optimal temperature for the enzyme cocktail was 40 °C, where hydrolytic activity peaked. Below 40 °C, hydrolysis efficiency positively correlated with temperature increase, while above this threshold, a significant inverse relationship was observed (Figure 8b).

**Figure 9:**
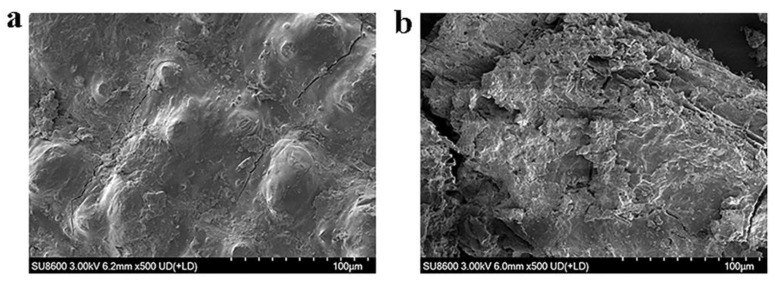
SEM micrographs of untreated and optimized enzyme cocktail-treated CDGs. (**a**) SEM micrographs of untreated CDGs. (**b**) SEM micrographs of CDGs treated with the optimized enzyme cocktail.

To further investigate the effect of the optimized enzyme cocktail on the structure of CDGs, scanning electron microscopy (SEM) and Fourier-transform infrared spectroscopy (FTIR) were used to characterize microstructural and molecular alterations before and after enzymatic treatment. As shown in Figure 9, SEM analysis revealed distinct morphological transformations. Untreated CDGs exhibited a compact surface structure with intact fiber bundles arranged in an ordered alignment and maintained their native extended conformation (Figure 9a). The enzymatic hydrolysis caused significant structural disruption, which led to increased surface roughness, enhanced porosity, and fiber breakage. Structural loosening was also observed (Figure 9b).

**Figure 10:**
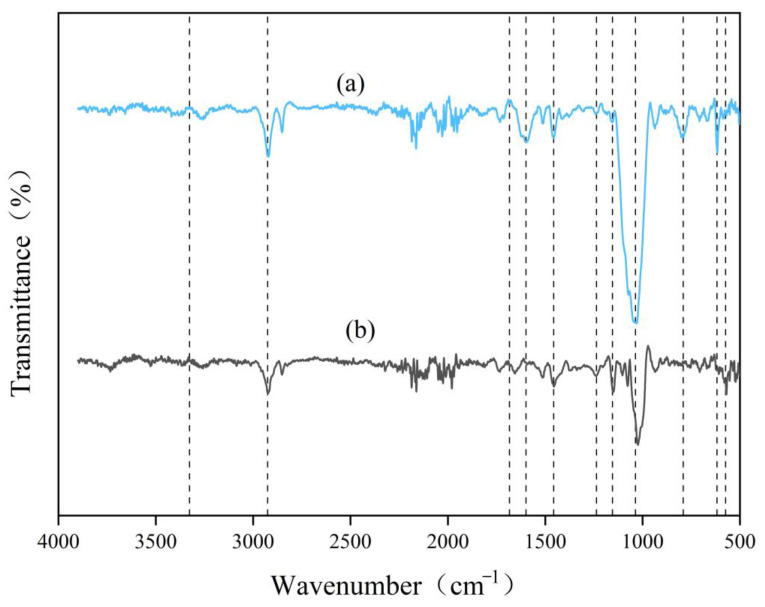
FTIR analysis of untreated and optimized enzyme cocktail-treated CDGs. (**a**) FTIR spectra of untreated CDGs. (**b**) FTIR spectra of optimized enzyme cocktail-treated CDGs.

The structural characteristics of CDGs were analyzed via FTIR to assess the degradation efficacy of the optimized enzyme cocktail (Figure 10). The broad absorption band around 3300 cm^−1^ was attributed to -OH stretching vibrations. The attenuation of this signal after enzymatic treatment indicated the disruption of intermolecular hydrogen bonds in the biomass [15,16]. Characteristic peaks at 2925 cm^−1^ (assigned to methyl and methylene groups) and those at 1236 cm^−1^, 1154 cm^−1^, 1036 cm^−1^, 791 cm^−1^, 576 cm^−1^, and 617 cm^−1^ (associated with functional groups of cellulose and hemicellulose) exhibited a reduction in intensity after enzymatic treatment [17,18], which indicated the partial degradation of these polysaccharides. The absorption bands at 1683 cm^−1^ and 1598 cm^−1^ corresponded to aromatic C=C stretching vibrations in lignin [19] and showed reduced intensity in the optimized enzyme cocktail-treated CDGs compared to in the untreated CDGs, which suggested the partial removal of lignin components.

## 3. Discussion

WRFs secrete an enzyme cocktail mainly composed of ligninolytic enzymes, cellulases, and hemicellulases to depolymerize lignocellulose into fermentable sugars, which serve as nutrients for fungal growth [1]. Agricultural waste, such as crop straws and CDGs, represents a critical biomass resource owing to its high lignocellulosic content. The biological conversion of these residues into value-added products, including biofeed, biochemicals, and biofuels [20], not only mitigates environmental pollution caused by agricultural waste but also alleviates resource scarcity [21]. However, the bottleneck in producing such bioproducts lies in the low saccharification efficiency and high costs of lignocellulose-degrading enzymes [4]. Consequently, identifying highly efficient enzyme cocktails for lignocellulose degradation is crucial to overcoming these challenges and advancing sustainable biorefinery processes.

The use of lignocellulosic biomass as cultivation substrates not only induces fungi to secrete highly active lignocellulose-degrading enzymes but also reduces enzyme production costs. For example, Li et al. [11] reported that using sugarcane bagasse as a substrate for *T. harzianum* K223452 resulted in an enzyme cocktail with a filter paper activity of 1.62 U/mL. Similarly, *P. ostreatus* grown on CDGs produced lignin-degrading enzymes with notable activities (Lac 152.68 U/g, Lip 15.56 U/g, MnP 0.34 U/g) and xylanase (10.98 U/g) [22]. Lignocellulose-degrading enzymes are inducible, and their activity and variety exhibit substrate-dependent variations. In this study, *C. trogii* Mafic-2001 cultivated on rice straw exhibited the highest laccase, cellulase, and xylanase activities compared to other biomass substrates, including oat straw, corn stover, corn cob, Napier grass, wheat bran, and rice straw. The observed variability in enzyme production across different biomass substrates might be attributed to differences in the compositional and structural characteristics of the biomass, particularly the lignocellulose content and complexity [23]. This substrate-dependent induction pattern aligned with previous studies. For example, Zhang et al. [14] reported that corn stover induced *T. harzianum* EM0925 to secrete more cellulase and xylanase compared with wheat bran, corn cob, sugarcane bagasse, and miscanthus. Similarly, steam-exploded wood residue significantly enhanced the xylanase, endoglucanase, and β-glucanase activities in *T. reesei* cultures compared with untreated wood residue [24]. Notably, structurally complex substrates might induce fungi to secrete enzyme cocktails with superior lignocellulose degradation efficiency [25]. In this study, the maximum cellulase and xylanase activities of *C. trogii* Mafic-2001 reached 3.29 U/mL and 25.83 U/mL, respectively, which surpassed those reported for other WRFs, including *Phanerochaete chrysosporium* (0.61 and 3.47 U/mL) [26], *Trametes versicolor* CTB863A (1.50 and 8.30 U/mL) [27], and *Trametes versicolor* (0.49 and 0.55 U/mL) [28]. These findings highlighted the potential of *C. trogii* Mafic-2001 as a robust producer of lignocellulose-degrading enzymes and made it a promising candidate for industrial biocatalytic applications.

The proteomic analysis of *C. trogii* Mafic-2001 induced by rice straw revealed that significant proportions of extracellular proteins were annotated to carbohydrate metabolic processes through GO and KEGG enrichment, which underscored its substantial potential for lignocellulose degradation. The complete deconstruction of lignocellulose requires the synergistic interplay of multiple enzymes [29]. In this study, the secretome of Mafic-2001 contained 287 CAZymes, which included 147 GHs, AAs, CEs, and PLs, which together formed the enzymatic foundation for efficient lignocellulose breakdown [8]. Notably, CBHs accounted for 7.57% of the secretome, and they hydrolyzed cellulose by progressively cleaving β-1,4-glycosidic bonds along cellulose chains to release cellobiose for further degradation by EGs and βGs [30,31,32]. This finding was consistent with previous reports, such as *Phanerochaete chrysosporium* secreting high-activity CBHs during tobacco straw and corn stover fermentation, which critically enhanced biomass degradation [33,34].

Furthermore, the Mafic-2001 secretome displayed a diverse array of hemicellulose-degrading enzymes, including backbone-cleaving enzymes (xylanases, xylosidases, and mannanases) and side-chain-targeting enzymes (galactosidases and α-L-arabinofuranosidases). Owing to the structural complexity of hemicellulose and its cross-linking with other plant cell wall components, the cooperative action of both backbone- and side-chain-degrading enzymes was crucial for efficient hydrolysis [5]. Additionally, Mafic-2001 secreted three carboxylesterases, which facilitated the degradation of cellulose and hemicellulose by hydrolyzing ester bonds and were recognized as crucial auxiliary enzymes [35]. In contrast to *Pycnoporus sanguineus*, which was reported to secrete only two carboxylesterases [36], Mafic-2001 exhibited a higher capacity for secreting these enzymes.

Laccases, a hallmark ligninolytic enzyme of WRFs [37], were prominently represented in the secretome, with five laccases identified (5.45% relative abundance). Compared with other WRFs, such as *Trametes versicolor* (three laccases) [38], *Stereum hirsutum* (one laccase) [38], *Peniophora lycii* (five laccases) [37], and *Trametes hirsuta* (two laccases) [37], Mafic-2001 ranks among the most prolific laccase producers reported to date. Intriguingly, lytic polysaccharide monooxygenases (LPMOs) of the AA9 family were also detected in the secretome of Mafic-2001. LPMOs are non-hydrolytic oxidative enzymes that have recently emerged as critical players in lignocellulose degradation [39,40]. LPMOs can degrade insoluble polysaccharides, such as crystalline cellulose and chitin [40], and can also co-degrade xyloglucan with glycoside hydrolases, thereby enhancing biomass conversion efficiency [5]. The presence of LPMOs, combined with the diverse enzymatic portfolio of Mafic-2001, further highlighted the strain’s superior hydrolytic performance.

The complete degradation of cellulose relies on the synergistic action of EGs, CBHs, and βGs [41]. Abundant EGs increase the frequency of random cleavage along cellulose backbones and generate additional sites for hydrolysis [42]. Meanwhile, βGs further hydrolyze cellobiose produced by CBHs into glucose and complete cellulose depolymerization [43]. The proteomic analysis of *C. trogii* Mafic-2001 revealed an imbalance in its cellulase system, with insufficient EG and βG activities relative to the high abundance of CBHs. This disparity might lead to cellobiose accumulation, which could trigger the product feedback inhibition of CBHs and ultimately reduce hydrolysis efficiency [10]. Enzymes derived from the same strain exhibit high synergies, mainly due to their co-evolution mechanism with substrate structure and similar physicochemical properties (e.g., optimal pH and temperature). The targeted supplementation of deficient enzymes, particularly those derived from the same fungal strain to maximize synergistic compatibility, has been proposed as a strategy to address such limitations [44,45]. In this study, the heterologous expression of *C. trogii* Mafic-2001-derived EGs and βGs in *P. pastoris* yielded recombinant enzymes that, when added to the native enzyme cocktail, enhanced the saccharification efficiency of CDGs by 172% compared with the effect obtained with the native enzyme cocktail alone. This enhancement aligned with prior strategies, such as those by Valadares et al. [13], who improved sugarcane bagasse cellulose degradation from 75% to 87% by augmenting *T. reesei* enzymes with white-rot fungal EGs and βGs. Similarly, the overexpression of EGs and βGs in *T. reesei* significantly improved cellulolytic efficiency [46,47]. These findings highlighted the critical role of balanced enzyme ratios in optimizing lignocellulose conversion and validated the effectiveness of tailored enzyme cocktails for industrial biomass processing. The current study employed a strategy of heterologously expressing target enzyme components and supplementing them into the native enzyme cocktail of *C. trogii* Mafic-2001 to enhance hydrolysis efficiency. While this approach demonstrated significant efficacy, the operational complexity associated with *P. pastoris* engineered strain fermentation and enzyme cocktail formulation may lead to increased costs. This research lays a crucial foundation for the preparation of enzyme cocktails, as subsequent studies could build upon these findings to explore more practical optimization strategies for improving enzymatic efficiency.

The appropriate ratio of hydrolytic enzymes within enzyme cocktails is crucial for achieving efficient hydrolysis [48]. In this study, the proportions of recombinant enzymes (rEG1 and rβG1) and the native enzyme cocktail Mafic-2001 were optimized using a mixture design. Multivariate regression analysis revealed that the native enzyme cocktail Mafic-2001 had the highest coefficient in the model, which highlighted its dominant contribution to hydrolysis efficiency. This finding was consistent with the analysis results of the Mafic-2001 secretome and previously reported characteristics of white-rot fungal enzyme cocktails, which were enriched with diverse CAZymes capable of synergistic lignocellulose degradation [49,50]. Notably, while the trace supplementation of rEG1 and rβG1 significantly enhanced hydrolysis efficiency, exceeding the threshold ratio (>0.1) resulted in a decline in performance. This phenomenon might arise from competitive enzyme–substrate binding or the saturation of the available substrate surface sites [51,52], which was similar to the inhibitory effects of enzyme excess reported by Tanaka and Du et al. in cellulase synergy studies [53,54]. This phenomenon underscored the importance of the precise regulation of enzyme ratios in industrial biocatalytic processes.

Further optimization revealed that at an enzyme loading of 320 mg/g, the reducing sugar yield reached 1.23 mg/mL, representing a significant improvement compared to pre-optimization levels. Through the holistic consideration of hydrolytic efficiency and enzyme cost, 320 mg/g was identified as the optimal enzyme loading [55]. Maximum saccharification efficiency was observed at pH 5.0 and 40 °C, which was consistent with the acidic optima typical of most white-rot fungal lignocellulose-degrading enzymes [56,57]. The structural transformation of CDGs after treatment with the optimized enzyme cocktail was further investigated through SEM and FTIR analysis. SEM observations revealed that enzymatic treatment caused the fibrous architecture to shift from a compact and ordered arrangement to a loose and porous morphology. Concurrent FTIR analysis demonstrated modifications in the lignocellulosic functional groups, accompanied by the partial removal of lignin, cellulose, and hemicellulose. These findings collectively suggested that the optimized enzyme cocktail effectively disrupted the lignocellulose through synergistic interactions, exposed the polysaccharide components, and enhanced their accessibility for hydrolysis [58]. Namnuch et al. investigated the structural modifications in sugarcane bagasse induced by enzymes derived from A. flavus KUB2 using SEM [59]. Similarly, Zeng et al. employed FTIR to analyze functional group alterations in wheat straw and specifically evaluated lignin methylation reactions [60]. Furthermore, Ye et al. characterized microstructural changes in treated paulownia wood through integrated FTIR and SEM analyses [61]. The application of these multimodal analytical approaches systematically assessed the enzymatic degradation efficiency of CDGs at the microscale and provided robust evidence for the synergistic degradation mechanisms targeting the lignin–carbohydrate complex in CDGs.

## 4. Materials and Methods

### 4.1. Strains, Plasmids, Reagents, and Lignocellulosic Biomass

The *C. trogii* Mafic-2001, *P. pastoris* X-33, and pPICZαA plasmid were kindly provided by Prof. Yunhe Cao of China Agricultural University. *Escherichia coli* Top 10 competent cells were purchased from Tiangen Biotech Co., Ltd. (Beijing, China). *Sac* I, *Eco*RI, and *Xba* I were obtained from TaKaRa (Kyoto, Japan). The substrate 2, 2′-azinobis-(3-ethylbenzothiazoline-6-sulfonic acid) (ABTS) was purchased from Beyotime (Shanghai, China). Sodium carboxymethyl cellulose (CMC) was purchased from Sigma-Aldrich (St. Louis, MO, USA). Beechwood xylan was purchased from Megazyme (Bray, Ireland). All other chemicals used in this study were of analytical grade.

The lignocellulosic biomass samples, including oat straw, corn stover, corn cob, Napier grass, wheat bran, and rice straw, were collected from Rongchang, Chongqing, China. CDGs were obtained from Wuliangye Group Co., Ltd. (Yibin, China).

### 4.2. Cultivation of C. trogii Mafic-2001 and Enzyme Activity Assay

Different carbon sources were used to induce enzyme production in *C. trogii* Mafic-2001. The fungus was aseptically transferred into an enzyme production medium containing 0.1% peptone, 0.14% ammonium sulfate, 0.2% KH_2_PO_4_, 0.03% CaCl_2_, 0.03% MgSO_4_·7H_2_O, 0.0005% FeSO_4_·7H_2_O, 0.00015% MnSO_4_·H_2_O, 0.00015% ZnSO_4_·7H_2_O, and 40 g of lignocellulosic biomass (including oat straw, corn stover, corn cob, Napier grass, wheat bran, or rice straw). The fermentation was conducted at 30 °C under aerobic agitation (220 rpm). Lignocellulosic enzyme activities in the culture solution were measured from day 2 to day 8 post inoculation. The optimal incubation time was determined based on the activities of cellulase, xylanase, and laccase.

Cellulase activity was measured using CMC as the substrate. A 1% (*w*/*v*) CMC solution was prepared in citrate–phosphate buffer (pH 5.5). One unit of cellulase activity was defined as the amount of enzyme required to release 1 μmol of reducing sugar per minute at 37 °C [62].

Xylanase activity was measured using the DNS method, as previously reported. A 1% (*w*/*v*) Beechwood solution was prepared in citrate–phosphate buffer (pH 5.5). One unit of xylanase activity was defined as the amount of enzyme needed to release 1 μmol of reducing sugar per minute at 37 °C [63].

Laccase activity was measured by using ABTS as the substrate (1 mM, pH 3.0). The culture supernatant (50 μL) was mixed with 950 μL of citrate–phosphate buffer (pH 3.0); 1 mL of ABTS solution (1 mM) was placed into another centrifuge tube and preheated at 30 °C for 3 min. The two solutions were then combined in a single centrifuge tube. The mixture was kept at 30 °C for 10 min, and the change in its absorbance at 420 nm was measured with a microplate reader. One unit of laccase activity was defined as the amount of enzyme that oxidized 1 μmol of ABTS per minute [64].

### 4.3. Preparation of Enzyme Cocktail Mafic-2001 and Extracellular Quantitative Proteome Analysis

*C. trogii* Mafic-2001 was cultured for 6 days in a medium containing rice straw as the enzyme-inducing substrate. The culture broth was then centrifuged at 10,000× *g* for 15 min at 4 °C, and the supernatant was collected. Ammonium sulfate precipitation was performed to prepare the enzyme cocktail, with a final concentration of 80% (*w*/*v*). After incubation at 4 °C for 2 h, the protein precipitates were pelleted by centrifugation under the same conditions (10,000× *g*, 4 °C, 15 min) and then resolubilized in sodium acetate buffer (50 mM, pH 5.0).

The protein solution was dialyzed using a dialysis membrane (3500 MW, Thermo Scientific™, Waltham, MA, USA) in specific phosphate-buffered saline (PBS, pH 7.4) at 4 °C. The PBS was replaced every 4 h until a total dialysis duration of 24 h was completed. The dialyzed protein solution was then subjected to freeze-drying to obtain the enzyme cocktail. The protein concentration was determined using the BCA Protein Assay Kit (Thermo Scientific™, Waltham, MA, USA), and the enzyme cocktail was stored at −20 °C for subsequent enzymatic analyses.

After the tryptic digestion of the Mafic-2001 enzyme cocktail, peptide desalting was performed using hydrophilic–lipophilic balanced solid-phase extraction cartridges. Peptide quantification was performed using a NanoDrop One spectrophotometer (Thermo Scientific™, Waltham, MA, USA). Peptides were analyzed via a liquid chromatography–tandem mass spectrometry (LC-MS/MS) system in DIA mode. Mass spectrometry data were processed using Spectronaut software (version 18, Biognosys AG, Zurich, Switzerland) against a customized fungal proteomic database, which integrated de novo genome-predicted fungal proteins and UniProt reference entries. The functional annotation of extracellular enzymes was then performed through GO term enrichment, KEGG pathway mapping, and carbohydrate-active enzyme (CAZy) classification, to systematically characterize enzymatic functionalities and metabolic roles.

### 4.4. Heterologous Expression of Cellulases from C. trogii Mafic-2001 in P. pastoris

#### 4.4.1. Cloning of Cellulase Gene from Mafic-2001 and Construction of Expressing Strains

Two pairs of primers were designed to amplify the *EG1* and *βG1* genes from Mafic-2001 based on the total DNA of *C. trogii* Mafic-2001. The primers were as follows: *EG1*-F 5′-ATGCCATCTTTCGCTGAGC-3′ and *EG1*-R 5′-TTATTTGCGCGCATTCGG-3′; *βG1*-F 5′-ATGTCGCGCGACTTCCTC-3′ and *βG1*-R 5′-TCACACCCCGTTCCATGTG-3′. The PCR products were ligated into the pGM-T vector and transformed into *E. coli* Top 10. Recombinant *E. coli* Top 10 was cultured on Luria–Bertani (LB) plates (0.5% NaCl, 0.5% yeast extract, 1% tryptone, and 1.5% agar) containing ampicillin (100 μg/mL). Recombinant plasmids (pGM-EG1 and pGM-βG1) were extracted from individual colonies and verified by Sanger sequencing using universal primers.

The *EG1* and *βG1* gene sequences were optimized based on the codon preference of *P. pastoris*. The optimized *EG1* and *βG1* sequences were synthesized by Tsingke Corp. (Beijing, China) and subjected to double digestion with *Eco*RI and *Xba*I. The resulting fragments were then ligated into the pPICZαA vector and transformed into *E. coli* Top 10. Transformed colonies were selected on LB plates supplemented with 100 µg/mL Zeocin™ and incubated at 37 °C until single colonies appeared. Individual colonies were inoculated into 50 mL flasks containing 10 mL LB liquid medium (100 µg/mL Zeocin™) and cultured overnight at 37 °C with shaking at 220 rpm. Recombinant plasmids (pPIC-EG1-opt and pPIC-βG1-opt) were then extracted using a plasmid extraction kit (Omega Bio-Tek, Norcross, GA, USA).

The plasmids pPIC-EG1-opt and pPIC-βG1-opt were linearized with *Sac*I and subsequently electroporated into *P. pastoris* X-33 (2000 V, 200 Ω, 5 ms). Transformed cells were plated on YPDS agar medium (1% yeast extract, 2% peptone, 2% glucose, 1 M sorbitol, 1.5% agar, and 100 µg/mL Zeocin™) for selection. Positive colonies harboring the recombinant constructs were isolated and designated as engineered strains expressing rEG1 and rβG1.

β-glucosidase activity was measured using p-nitrophenyl-β-D-glucopyranoside (pNPG) (Shanghai Yuanye Bio-Technology Co., Ltd., Shanghai, China) as the substrate. A 5 mM pNPG solution was prepared in citrate–phosphate buffer (pH 5.0). One unit of β-glucosidase activity was defined as the amount of enzyme required to release 1 μmol of p-nitrophenol per minute.

#### 4.4.2. Preparation of Recombinant rEG1 and rβG1 Samples

Positive clones of X-33/EG1 and X-33/βG1 were selected and cultured in 50 mL of buffered glycerol-complex (BMGY) medium (1% yeast extract, 2% peptone, 100 mM potassium phosphate, pH 6.0, 1.34% YNB, 4 × 10^−5^% biotin, 1.00% glycerol) at 28 °C and 220 rpm for 24 h. The cells were collected by centrifugation at 5000 rpm for 5 min at 4 °C. The cell pellet was re-suspended in 50 mL of buffered methanol-complex (BMMY) medium (1% yeast extract, 2% peptone, 100 mM potassium phosphate, pH 6.0, 1.34% YNB, 4 × 10^−5^% biotin, 0.5% methanol) and cultured at 28 °C with 220 rpm agitation for 96 h. Methanol (0.5%, *v*/*v*) was added to the culture every 12 h to induce protein expression. The detailed operation procedure was reported by Bao et al. [64]. After fermentation, the supernatant was collected, and a small portion was concentrated using a Vivacon^®^ 500 (SARTORIUS, Gottingen, Germany) ultrafiltration tube. rEG1 and rβG1 were analyzed by SDS-PAGE. The rEG1 and rβG1 samples were obtained by freeze-drying most of the supernatant. The protein concentration was determined using a BCA protein detection kit, and the proteins were stored at −20 °C for later use.

### 4.5. Formulation of the Enzyme Cocktail Mafic-2001 and Optimization of Enzymatic Hydrolysis Conditions for CDGs

CDGs were dried at 80 °C for 24 h, pulverized, and sieved through a 40-mesh sieve. Subsequently, 200 mg of the processed CDGs was dissolved in 10 mL of Na_2_HPO_4_–citrate buffer (pH 4.0) at a solid-to-liquid ratio of 1:50 (*w*/*v*). The mixture was transferred to a 50 mL centrifuge tube containing the enzyme and incubated in a rotary shaker at 200 rpm for enzymatic hydrolysis. After centrifugation to separate the supernatant and solid residue, the reducing sugar content in the hydrolyzed supernatant was quantified using the 3,5-dinitrosalicylic acid (DNS) method. A reaction mixture containing 80 μL of distilled water, 80 μL of sample, and 200 μL of DNS was prepared, boiled at 100 °C for 5 min, and rapidly cooled to room temperature. The total volume was adjusted to 1 mL by adding 640 μL of distilled water. Absorbance was measured at 540 nm using a microplate reader, and concentrations were calculated against a glucose-derived standard curve.

The sample was mixed with DNS reagent (1:1 *v*/*v*), boiled at 100 °C for 5 min, and cooled to room temperature. Absorbance was measured at 540 nm. The reducing sugar concentration was determined using a glucose standard curve.

First, the hydrolysis efficiency of CDGs was systematically evaluated using different enzyme combinations. The experimental groups included the native enzyme cocktail Mafic-2001, rEG1, rβG1, Mafic-2001–rEG1 (1:1, *w*/*w*), Mafic-2001–rβG1 (1:1, *w*/*w*), rEG1–rβG1 (1:1, *w*/*w*), and a ternary combination of Mafic-2001–rEG1–rβG1 (1:0.5:0.5, *w*/*w*). Enzyme loading was set at 30 mg enzyme protein per gram of substrate (30 mg/g, *w*/*w*). Reactions were conducted in triplicate by incubating the mixtures in a shaking incubator at 200 rpm and 40 °C for 24 h. Samples were collected at 12 h intervals for reducing sugar content analysis.

According to the aforementioned results, the optimal enzyme combination was selected for further optimization. A simplex-lattice mixture design comprising 13 experimental runs was implemented using Design-Expert 12.0 software (Stat-Ease, Minneapolis, MN, USA). The relative proportions of the three enzymes in the combinatorial system were defined as independent variables, with reducing sugar yields from the hydrolysis of CDGs serving as the response variable. The minimum value of the three enzymes was set to 0.05, and the sum of the three enzymes was 1.0. A response surface methodology was employed to fit the experimental data and identify the best-performing regression model for predicting the optimal enzyme ratios. The detailed procedure was reported by Du et al. [65]. Following the determination of the optimal ratio of the three enzymes through regression modeling, an enzyme cocktail formulated at this optimized ratio was employed to hydrolyze Chinese distillers’ grains (CDGs), and validation experiments were subsequently conducted.

According to the identification of the optimal enzyme combination and ratio, the concentration-dependent hydrolysis efficacy of the formulated enzyme cocktail on CDGs was investigated. Enzyme loadings were tested at various concentrations (80, 120, 160, 200, 240, 280, 320, and 360 mg/g). Hydrolysis reactions were performed in a shaker at 200 rpm for 24 h. The concentration of reducing sugars in the supernatant was quantified to evaluate enzymatic performance.

Finally, under the optimal ratio and dosage of the optimized enzyme cocktail, the optimal pH and temperature for degrading CDGs were investigated. To determine the optimal pH, the enzyme cocktail and CDGs were incubated in buffer solutions with pH values of 4.0, 4.5, 5.0, 5.5, 6.0, 6.5, 7.0, and 8.0 while maintaining other reaction parameters constant. The reducing sugar content in the hydrolysate was then analyzed. For temperature optimization, enzymatic reactions were conducted in a shaker at 200 rpm, with temperatures set at 30 °C, 35 °C, 40 °C, 45 °C, 50 °C, 55 °C, 60 °C, and 65 °C. The reducing sugar content in the supernatant was quantified to evaluate enzymatic performance.

### 4.6. Characterization of CDGs Before and After Enzymatic Hydrolysis

The untreated and treated CDGs were dehydrated using an air-dry oven (Shanghai Yiheng Scientific Instrument Co., Ltd., Shanghai, China). A scanning electron microscope (SU8600, Hitachi, Japan) was employed to characterize the changes in the surface morphology of the untreated and enzyme-treated CDGs. The CDGs were sputter-coated with gold and photographed at a magnification of 500×.

An FTIR spectrometer (Nicolet iN10, Thermo Scientific™, Waltham, MA, USA) and KBr pellet preparation were used to investigate the chemical structure changes in the untreated and enzyme-treated CDGs in the range of 500–4000 cm^−1^.

## 5. Conclusions

The use of rice straw as a carbon source in the culture medium significantly enhanced the secretion of lignocellulose-degrading enzymes by *C. trogii* Mafic-2001. This fungal strain exhibited exceptional production of ligninolytic enzymes, along with cellulase and hemicellulase that worked synergistically. To address the limitation of low enzymatic efficiency caused by imbalanced cellulase ratios in the native enzyme cocktail, recombinant enzymes rEG1 and rβG1 were successfully expressed heterologously in *P. pastoris* X-33. A novel enzyme cocktail (rEG1–rβG1–Mafic-2001 = 0.05:0.09:0.86) was developed. The optimized enzyme cocktail demonstrated significantly improved degradation efficiency for CDGs under optimal conditions. This study highlights the multi-enzyme synergy of the Mafic-2001 enzyme cocktail in lignocellulose biomass degradation and establishes an efficient enzyme cocktail for CDG hydrolysis through the targeted supplementation of rate-limiting components. At the same time, it also provides a strategy for the preparation of efficient lignocellulosic enzyme cocktails, thus laying the groundwork for the sustainable valorization of lignocellulosic biomass.

## Figures and Tables

**Table 1:** Cellulases and their abundance in the proteome of *C. trogii* Mafic-2001.

Predicted Protein	Accession Number/NR	CAZy Domain	Abundance (%)
Exoglucanase	KAI0800646.1	GH7	4.622
Exoglucanase	RPD55895.1	GH7	2.302
Exoglucanase	PAV23805.1	GH6-CBM1	0.411
Exoglucanase	RPD64364.1	GH7	0.243
Endo-1,4-β-glucanase	KAI0800008.1	GH61	1.496
Endo-1,4-β-glucanase	KAJ8463859.1	GH61	1.318
Endo-1,4-β-glucanase	KAI0751843.1	GH61	0.065
Endo-1,4-β-glucanase	RPD63359.1	GH16	0.241
Endo-1,4-β-glucanase	KAI0371965.1	GH12	0.170
Endo-1,4-β-glucanase	KAI0691154.1	GH12	0.023
Endoglucanase	RPD54862.1	GH5-CBM1	1.404
Endoglucanase	KAI0801045.1	GH5-CBM1	0.147
Endoglucanase	RPD65704.1	GH5	0.014
β-glucosidase	KAI0630095.1	GH55	0.404
β-glucosidase	KAI0719308.1	GH5	0.006
β-glucosidase	KAI0691695.1	GH3	0.170
β-glucosidase	TFK87958.1	GH3	0.068
β-glucosidase	RPD63079.1	GH3	0.051
β-glucosidase	RDX41311.1	GH3	0.038
β-glucosidase	OJT12611.1	GH3	0.016
β-glucosidase	KAI0332110.1	GH55	0.018
β-glucosidase	PIL27667.1	GH1	0.017
β-glucosidase	RDX56565.1	GH5	0.016
β-glucosidase	KAI0719308.1	GH5	0.006

**Table 2:** Hemicellulases and their abundance in the proteome of *C. trogii* Mafic-2001.

Predicted Protein	Accession Number/NR	CAZy Domain	Abundance (%)
Endo-1,4-β-xylanase	KAG8859488.1	GH10	0.436
Endo-1,4-β-xylanase	TFK88994.1	GH10-CBM1	0.066
Endo-1,4-β-xylanase	KAJ8496589.1	GH10-CBM1	0.021
Xyloglucanase	KAI0724491.1	GH74-CBM1	0.400
β-xylosidase	RPD54124.1	GH3	0.278
β-xylosidase	XP_007370314.1	GH5	0.193
β-mannosidase	KAI0723105.1	GH2	0.182
β-mannosidase	TFK90368.1	GH2	0.009
Endo-1,4-β-mannosidase	KAI0756630.1	GH5	0.084
Endo-1,4-β-mannosidase	KAJ8468592.1	GH5	0.023
Endo-1,6-α-mannosidase	KAI0702396.1	GH76	0.039
α-1,2-mannosidase	RPD61256.1	GH47	0.049
α-mannosidase	RPD58628.1	GH38	0.020
α-galactosidase	RPD81798.1	GH27	0.142
β-galactosidase	TFK88994.1	GH35	0.066
β-galactosidase	KAI0747487.1	GH35	0.023
Carboxylesterase	KAI0705476.1	CE1	0.036
Carboxylesterase	KAI0711489.1	CE1	0.033
Carboxylesterase	ROV98550.1	CE1	0.009

**Table 3:** Lignin-degrading enzymes and their abundance in the proteome of *C. trogii* Mafic-2001.

Predicted Protein	Accession Number/NR	CAZy Domain	Abundance (%)
Laccase	AAF70119.2	AA1	3.384
Laccase	ABD93940.1	AA1	1.918
Laccase	CAL23367.1	AA1	0.094
Laccase	KAI0714945.1	AA1	0.027
Laccase	AMJ39540.1	AA1	0.024
Manganese peroxidase	KAI0374001.1	AA2	0.039

**Table 4:** Redox-active enzymes and their abundance in the proteome of *C. trogii* Mafic-2001.

Predicted Protein	Accession Number/NR	Abundance (%)
FAD-binding oxidoreductase	KAI0765219.1	0.335
Alcohol oxidase 1	KAI0717593.1	0.006
Aryl-alcohol dehydrogenase	KAI0716463.1	0.037
Aryl-alcohol dehydrogenase	KAI0767476.1	0.018
Aryl-alcohol dehydrogenase	KAJ8475436.1	0.010
Oxidoreductase	KAI0779384.1	0.002
Oxidoreductase	KAI0699741.1	0.002
GMC oxidoreductase	KAI0773689.1	0.120
GMC oxidoreductase	KAI0655366.1	0.115
Peroxiredoxin	KAI0739514.1	0.066
Peroxiredoxin	KAI0773839.1	0.010
Peroxiredoxin	OSC98867.1	0.009
Alcohol dehydrogenase	KAI0716463.1	0.037
Lytic polysaccharide monooxygenase	KAI0743742.1	0.271

**Table 5:** Pectin-degrading enzymes and their abundance in the proteome of *C. trogii* Mafic-2001.

Predicted Protein	Accession Number/NR	CAZy Domain	Abundance (%)
Rhamnogalacturonan Acetylesterase	KAI0770387.1	CE12	0.904
Endo-polygalacturonase	RDX42877.1	GH28	0.196
Exopolygalacturonase	KAI0740761.1	GH28	0.031
Exopolygalacturonase	RPD64593.1	GH28	0.017
Exopolygalacturonase	KAI0760371.1	GH28	0.027
Polygalacturonase	RPD79321.1	GH28	0.010
α-L-rhamnosidase	KAI0735548.1	GH28	0.009

**Table 6:** Starch-degrading enzymes and their abundance in the proteome of *C. trogii* Mafic-2001.

Predicted Protein	Accession Number/NR	CAZy Domain	Abundance (%)
Glucoamylase	RPD62695.1	GH15-CBM20	0.640
α-amylase	KAI0806620.1	GH13	0.029
α-amylase	KAI0362747.1	GH13	0.017
α-amylase	RDX41646.1	GH13-CBM20	0.010

**Table 7:** Reducing sugar yield as influenced by enzyme ratios in the mixture design.

Relative Ratio of Enzymes			Reducing Sugar (mg/mL)
Mafic-2001	rβG1	rEG1	
0.333	0.333	0.333	0.363
0.192	0.192	0.617	0.312
0.900	0.050	0.050	0.429
0.475	0.475	0.050	0.393
0.050	0.475	0.475	0.268
0.050	0.900	0.050	0.250
0.475	0.050	0.475	0.355
0.192	0.617	0.192	0.295
0.617	0.192	0.192	0.394
0.050	0.050	0.900	0.273

## Data Availability

The datasets used and/or analyzed during the current study are available from the corresponding author upon reasonable request.

## Supplementary Materials

The following supporting information can be downloaded at https://www.mdpi.com/article/10.3390/ijms26104702/s1.

## Abbreviations

The following abbreviations are used in this manuscript:

EG	Endoglucanase
βG	β-glucosidases
CBH	Cellobiohydrolase
CDGs	Chinese distillers’ grains
WRFs	White-rot fungi
*C. trogii*	*Coriolopsis trogii*
*P. pastoris*	*Pichia pastoris*
CMCase	Carboxymethyl cellulase
GO	Gene Ontology
KEGG	Kyoto Encyclopedia of Genes and Genomes
CAZymes	Carbohydrate-active enzymes
GHs	Glycoside hydrolases
AAs	Auxiliary activities
CBMs	Carbohydrate-binding modules
CEs	Carbohydrate esterases
GTs	Glycosyltransferases
PLs	Polysaccharide lyases
LPMO	Lytic polysaccharide monooxygenase
CAI	Codon adaptation index
ABTS	2,2′-azinobis-(3-ethylbenzothiazoline-6-sulfononic acid)
CMC	Sodium carboxymethyl cellulose
DNS	3,5-dinitrosalicylic acid
SEM	Scanning electron microscopy
SDS-PAGE	Sulphate–poly-acrylamide gel electrophoresis
