# Supplementary material for: An Investigation of the Secretome Composition of Coriolopsis trogii Mafic-2001 and the Optimization of the Mafic-2001 Enzyme Cocktail to Enhance the Saccharification Efficacy of Chinese Distillers’ Grains
Source: Int J Mol Sci. 2025 May 14;26(10):4702. doi: 10.3390/ijms26104702 (PMC12111451; doi:10.3390/ijms26104702)
Supplement: Supplementary file 1 [file ijms-26-04702-s001.zip › ijms-3608828-supplementary.pdf]

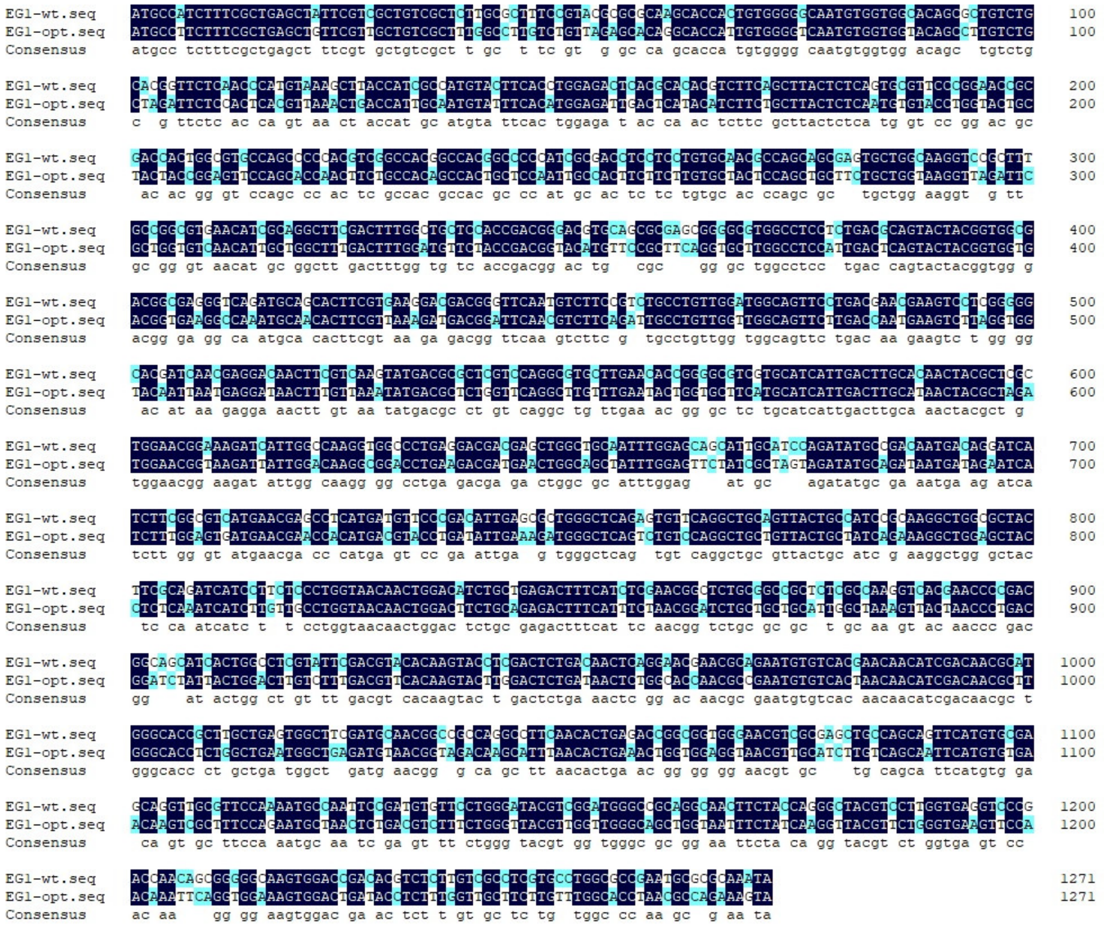


**Figure S1.** Alignment of wild type EG1 gene (EG1-wt) sequence and optimized type EG1 gene (EG1-opt) sequence.

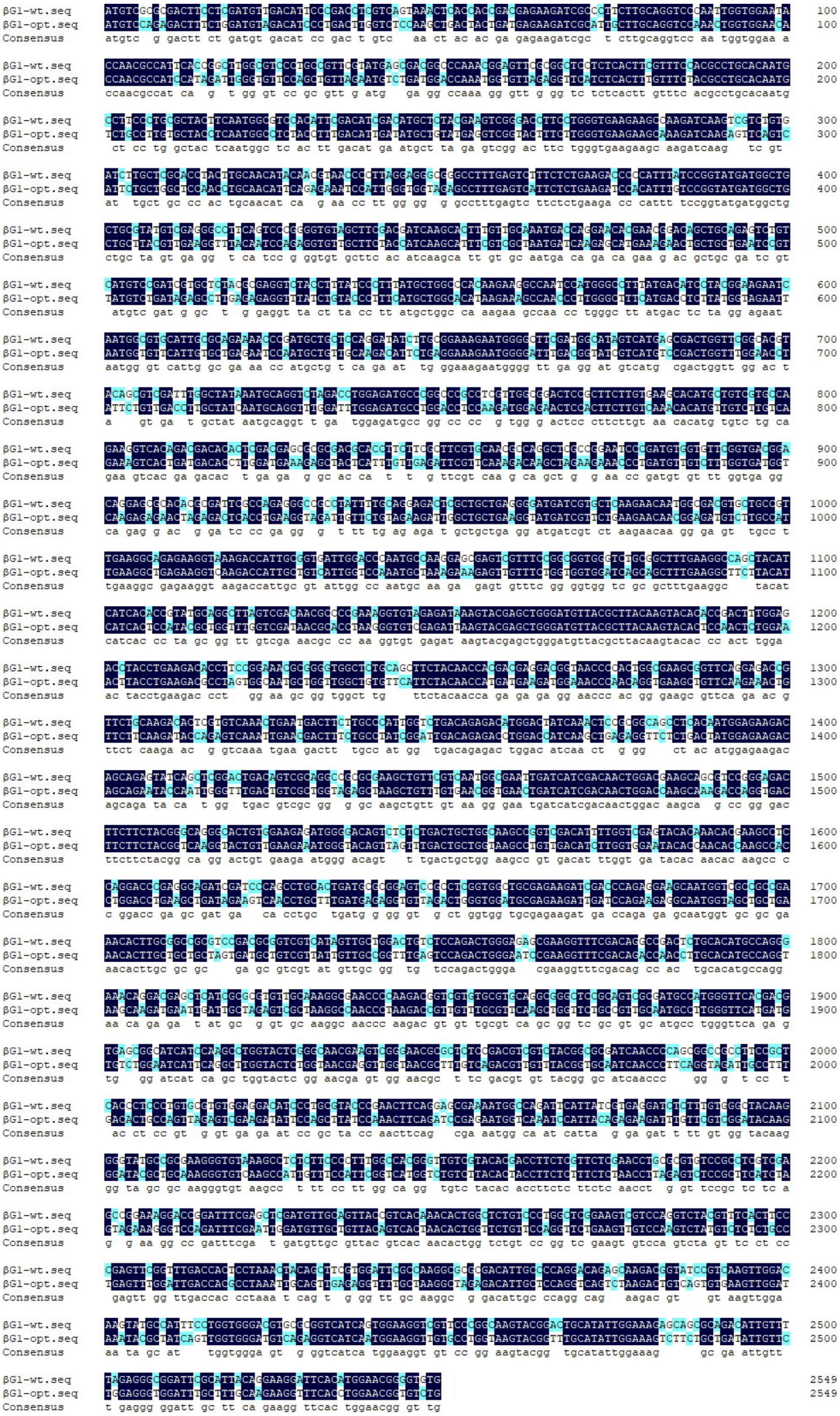

**Figure S2.** Alignment of wild type β G1 gene (βG1-wt) sequence and optimized type EG1 gene (βG1-opt) sequence.

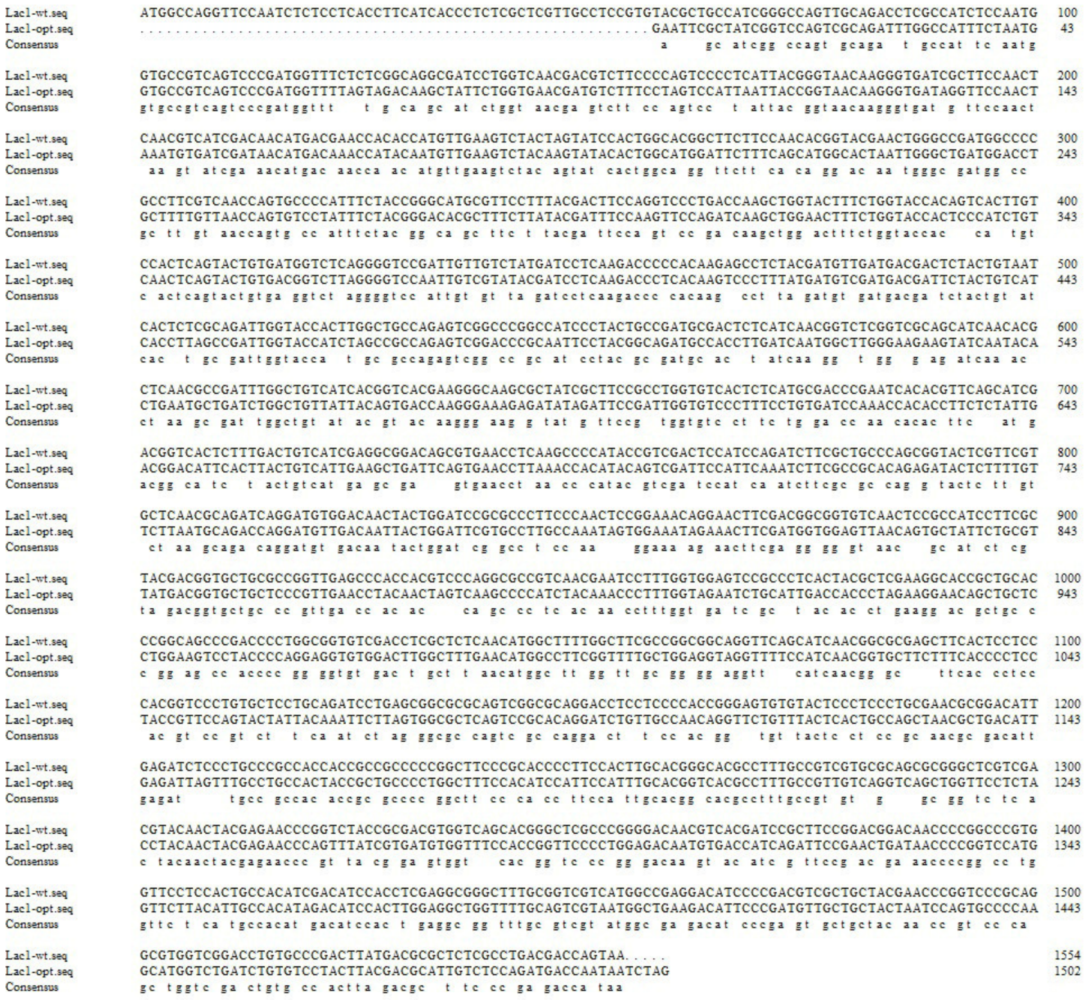

**Figure S3.** Alignment of wild type *Lac1* gene (*Lac1*-wt) sequence and optimized type *Lac1* gene (*Lac1*-opt) sequence

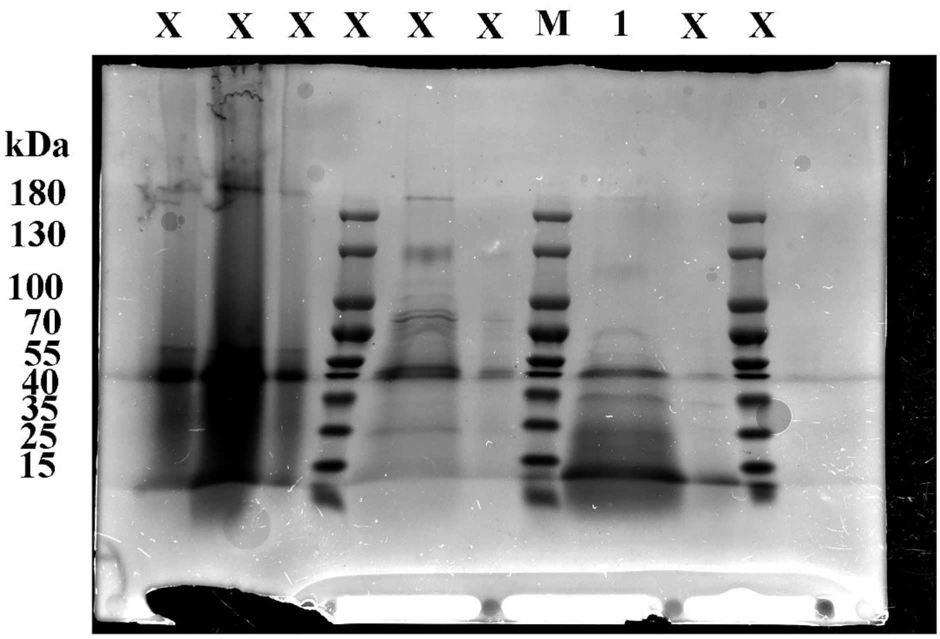


**Figure S4.** The original gel image of SDS-PAGE analysis for the protein expressed by the recombinant strain X-33/EG1 (Figure 5a). M: Protein molecular marker; 1. supernatant from recombinant strain X-33/EG1 in shake flasks. X: Lanes not included in the final figure.

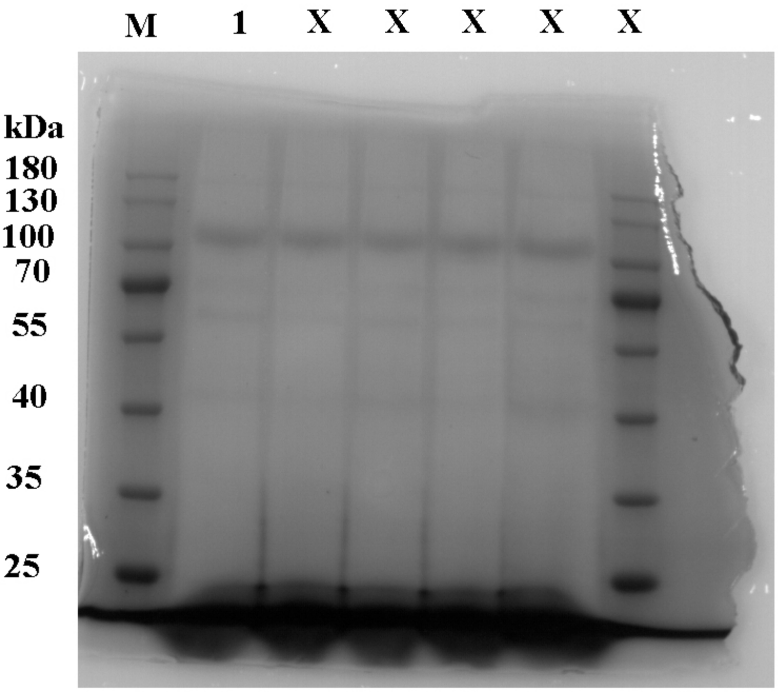


**Figure S5.** The original gel image of SDS-PAGE analysis for the protein expressed by the recombinant strain X-33/βG1 (Figure 5b). M: Protein molecular marker; 1. supernatant from recombinant strain X-33/βG1 in shake flasks. X: Lanes not included in the final figure.
